# The Structure and Composition of Extracted Pectin and Residual Cell Wall Material from Processing Tomato: The Role of a Stepwise Approach versus High-Pressure Homogenization-Facilitated Acid Extraction

**DOI:** 10.3390/foods10051064

**Published:** 2021-05-12

**Authors:** Jelle Van Audenhove, Tom Bernaerts, Victor De Smet, Sophie Delbaere, Ann M. Van Loey, Marc E. Hendrickx

**Affiliations:** Laboratory of Food Technology and Leuven Food Science and Nutrition Research Centre (LFoRCe), Department of Microbial and Molecular Systems (M2S), KU Leuven, Kasteelpark Arenberg 22, Box 2457, 3001 Leuven, Belgium; tom.bernaerts@kuleuven.be (T.B.); VictorDS97@outlook.com (V.D.S.); sophie.delbaere@kuleuven.be (S.D.); ann.vanloey@kuleuven.be (A.M.V.L.); marceg.hendrickx@kuleuven.be (M.E.H.)

**Keywords:** processing tomato, cell wall polysaccharides, pectin extraction methods, cell wall interactions, high-pressure homogenization, structural characterization

## Abstract

In literature, different pectin extraction methods exist. In this study, two approaches starting from the alcohol-insoluble residue (AIR) of processing tomato are performed in a parallel way to facilitate the comparison of pectin yield and the compositional and structural properties of the extracted pectin and residual cell wall material obtained. On the one hand, pectin is extracted stepwise using hot water, chelating agents and low-alkaline conditions targeting fractionation of the pectin population. On the other hand, an industrially relevant single-step nitric acid pectin extraction (pH 1.6) is performed. In addition to these conventional solvent pectin extractions, the role of high-pressure homogenization (HPH) as a physically disruptive treatment to facilitate further pectin extraction from the partially pectin-depleted fraction obtained after acid extraction is addressed. The impact of HPH on the pectin cell wall polysaccharide interactions was shown as almost two thirds of the residual pectin were extractable during the subsequent extractions. For both extraction approaches, pectin obtained further in the sequence was characterized by a higher molecular mass and a higher amount of rhamnogalacturonan I domains. The estimated hemicellulose and cellulose content increased from 56 mol% for the AIR to almost 90 mol% for the final unextractable fractions of both methods.

## 1. Introduction

Plant cells are surrounded by a cell wall, which is a supramolecular assembly of associating polysaccharides, namely cellulose, hemicellulose and pectin, and proteins [1,2,3]. Cellulose has a crystalline structure since its glucan chains (β-1,4-linked) can aggregate into microfibrils because of their linear nature [2]. However, after the synthesis of the glucan chain, the aggregation is often incomplete, which results in amorphous regions providing the semi-crystalline properties of cellulose [4]. Hemicellulose encompasses a group of polysaccharides with diverse structures, such as xyloglucans, xylans and mannans [5]. Xyloglucan is the main hemicellulose in the primary cell wall of edible fruits and vegetables of dicotyledonous plants [5]. Xyloglucan consists of a backbone of β-1,4-linked glucose (Glc) units to which xylose (Xyl) residues are bound [6]. Pectin is a complex galacturonic acid (GalA)-rich polymer consisting of three main domains, namely, homogalacturonan (HG) and rhamnogalacturonan I (RG-I) and II (RG-II) [7]. HG is a linear polymer consisting only of α-1,4-linked GalA units which can be methyl-esterified on C-6 and O-acetylated on O-2 or O-3 [8,9]. The percentage of GalA units that are methyl-esterified is expressed as the degree of methyl esterification (DM) [7]. RG-I consists of a backbone of repeating α-1,2-linked-rhamnose-α-1,4-GalA and has side chains on 20–80% of the rhamnose (Rha) units, which are mainly composed of galactose (Gal) and arabinose (Ara) [7,9]. RG-II has a backbone of GalA units (α-1,4-linked) substituted with four types of side chains constituted by 12 different sugars covering some complex residues [8,9].

Over the past decades, the precise architecture of plant cell walls has been extensively studied. Hayashi [10] and Fry [6] proposed that xyloglucan can bind to the cellulose microfibrils surface as well as be physically entrapped in the microfibrils, suggesting the role of xyloglucan in the crosslinking of cellulose. In this traditionally most popular model, the pectin network forms a matrix which is physically entangled with the cellulose–xyloglucan network often called the “tethered network” and with the protein network, which together constitute plant cell walls [11,12]. However, this traditional model has been revised by many studies. Popper and Fry [13] proposed that RG-I is linked to xyloglucan as they showed for different angiosperms (including tomato) that a substantial fraction of xyloglucan was linked to an acidic polymer. This is in accordance with the cell wall model presented by Keegstra et al. [14] in which the side chains of RG-I are attached to the reducing ends of xyloglucan. Interactions between cellulose and pectin, especially with the neutral sugar side chains of the RG-I region, have also been shown to exist [15,16]. Moreover, the existence of xyloglucan tethers between cellulose microfibrils has been revisited. Instead of forming tethers, the fraction of xyloglucan involved in the formation of the load-bearing network is recently suggested to be intertwined with cellulose leading to ‘biomechanical hotspots’, which are close contact points between the cellulose microfibrils mediated by xyloglucan [17,18]. Although on field emission scanning electron microscopy images, fibrils crosslinking cellulose microfibrils were visualized, they were, based on the use of specific enzymes, suggested to be cellulose instead of xyloglucan [19].

As an ingredient, pectin has found many applications in food industry, where it is used especially as a gelling agent in the production of jams and jellies but also as a thickener and texturizer [20]. Commercially, pectin is obtained by extraction from a limited range of sources, of which citrus peel (85.5%) is the most common, followed by apple pomace (14.0%) and sugar beet pulp, which only accounts for 0.5% [7,21]. Most commonly, extraction of pectin is performed using mineral acid solutions, mainly because of the high yield and the low cost of this method [7]. The most important factors determining the pectin yield are the pH of the medium, extraction time and temperature [22,23] which are commonly chosen within the following ranges: 1.5–3, 0.5–6 h and 60–100 °C, respectively [24]. Generally, higher yields are found when harsher conditions, i.e., lower pH, longer extraction time and higher temperature, are applied [22,23]. Since the linkages in the neutral sugar side chains are more acid-labile than those of the pectin backbone [25], these harsh conditions have been shown to predominantly degrade the side chains of pectin, arabinan in particular, thus leading to extracted pectin enriched in GalA [22,23].

Other solvents like (hot) water, water containing chelating agents and alkaline extraction media can be used to extract different pectin fractions from a fruit or vegetable material [26]. When these extractions are performed in this stepwise manner, pectin can be extracted selectively based on the interactions involved [7]. Pectin extraction with water yields loosely bound pectin [26,27]. The use of higher temperature results in a higher pectin extraction yield as shown in studies on Japanese plum, apple and sugar beet [28,29]. Water containing a chelating compound, e.g., cyclohexane-trans-1,2-diaminetetraacetic acid (CDTA), disturbs the pectin interaction by Ca^2+^ bridges and thus allows extraction of pectin with lower DM [26,30,31]. Finally, extraction of pectin with diluted alkali, e.g., 0.05–0.1 M Na_2_CO_3_ or 0.1 M NaOH, mainly yields pectin which interacts with other cell wall polysaccharides by low-alkali-labile bonds, such as ester and hydrogen bonds [30,31,32,33,34,35]. 

A possible strategy to improve pectin extractability is by use of physical methods. Studies have shown that high-pressure homogenization (HPH) could increase the amount of pectin that could be extracted with hot water from the alcohol-insoluble residue (AIR) of orange pulp fibers and carrot when the material was (in the suspended state) subjected to HPH because of its disruptive effect leading to weakening of the interactions of pectin in the cell wall [36,37]. The effect of HPH seemed to be dependent on the matrix studied since this increase in hot water extractability was not observed for broccoli [38]. For tomato, the changes in pectin extractability due to HPH seemed dependent on the pretreatment of the mixed tomato tissue since for raw tomato, no changes were observed, whereas for high-temperature blanched tomato, more pectin became chelator-extractable at the expense of low-alkaline-extractable pectin [39]. The effect of another physical method, namely, ball milling, on the interactions between cell wall polymers and their extractability was studied by Broxterman and Schols [40]. Ball milling of dry chelating agent-unextractable solids of carrot, tomato and strawberry was shown to assist in the subsequent solubilization of cell wall polymers, namely, pectin, for all matrices, but also hemicellulose, especially in the case of the latter two matrices [40].

The starting material of the current study is tomato, which is a relevant matrix as it has a very high worldwide production (around 159 million tons per year) [41]; also a considerable amount of tomato pomace waste is generated by the tomato processing industry [42]. Since currently the demand for pectin is high and increasing, the possibility to use other sources such as tomato pomace next to citrus peel, apple pomace and sugar beet being the current commercial pectin sources should be investigated [43]. The main goal of this study was to compare not only the pectin extraction yield and the chemical structural properties of the extracted pectin, but also the polysaccharide composition of the unextractable cell wall material (CWM) obtained during and after two extensive pectin extraction approaches performed on processing tomato tissue AIR. More specifically, on the one hand, a stepwise extraction procedure consisting of consecutive extractions with hot water, with CDTA as a chelating compound and with a low-alkaline medium was performed. Following this approach, pectin fractionation based on extractability under specific conditions was expected. On the other hand, acid pectin extraction, which is representative for industrial extraction, was included. After partial pectin depletion by this acid extraction, the residual CWM fraction was subjected to HPH in the suspended state to address whether this physically disruptive action by HPH could improve the extractability of the residual pectin during water and repeated acid extraction performed after HPH. To the best of our knowledge, no research has been reported so far on the effect of HPH on the extractability of pectin from a CWM fraction which was already partially pectin-depleted by the same extraction. The parallel experimental design of the two extraction approaches starting on tomato AIR facilitates evaluation and comparison of the differences in the extraction efficiency of each method and the structural characteristics of the extracted pectin. As a consequence, the results obtained could serve as a starting point in selecting appropriate extraction conditions aiming at certain tomato pectin structures or even the residual material after extraction for more applied research on its functional properties and applications.

## 2. Materials and Methods

### 2.1. Materials

Throughout this work, the San Marzano tomato (*Solanum lycopersicum*) variety, a plum type tomato, was used. These processing tomatoes were bought at a local shop in Belgium and kept at room temperature for up to five days before blanching. All the tomatoes used were similar in ripeness based on visual perception of their redness. 

Technical ethanol (99%), technical acetone, isopropanol, Na_2_CO_3_ and NaOH pellets were obtained from VWR (Leuven, Belgium). Cyclohexane-trans-1,2-diaminetetraacetic acid monohydrate was obtained from Tokyo Chemical Industry (Zwijndrecht, Belgium). HCl, H_2_SO_4_ (concentration ≥ 95%) and disodium tetraborate decahydrate were bought from Fisher Scientific (Merelbeke, Belgium). H_2_SO_4_ (72% *w*/*w*) was obtained from Alfa Aesar (Kandel, Germany) and NaOH (50% *w*/*w*) was obtained from J.T. Baker (Gliwice, Poland). Rhamnose monohydrate, LiOH, acetic acid and 3-phenylpenol, potassium acetate, nitric acid and NaNO_3_ were bought from Acros Organics (Geel, Belgium). Galacturonic acid monohydrate and fucose were obtained from Sigma-Aldrich (Diegem, Belgium), arabinose from Fluka Biochemika (Buchs, Switzerland), mannose from Fluka Analytical (Buchs, Switzerland), glucose monohydrate from Riedel-de-Haën (Seelze, Germany), xylose from UCB (Leuven, Belgium) and galactose from Merck (Darmstadt, Germany). Ultrapure water (organic free, 18.2 MΩ∙cm resistance) used in some of the procedures was provided by a Simplicity^TM^ 150 system (Millipore, Billerica, MA, USA).

### 2.2. Blanching of Tomatoes

To avoid enzyme activity (e.g., pectin methyl esterase or polygalacturonase) in any further steps performed on the tomato tissue, the red-ripe tomatoes were blanched [39]. For this, the washed tomatoes were cut in slices of approximately 5 mm-thick which were positioned next to each other in a plastic bag, vacuum-packed and consequently blanched for 8 min at 95 °C in a water bath. After this blanching step, the bags were immersed in an ice bath and afterwards frozen with liquid N_2_. The blanched tomato slices were kept at −40 °C until further use.

### 2.3. Generation of the Alcohol-Insoluble Residue and Cell Wall Material Fractions

**Figure 1:**
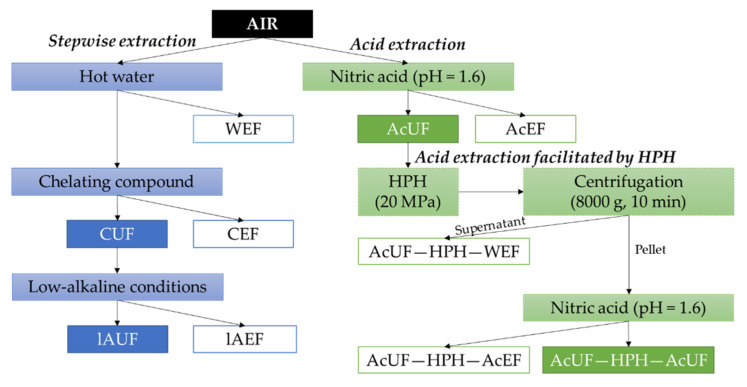
Schematic overview of the extraction methods starting from the alcohol-insoluble residue (AIR) and the obtained unextractable fractions (UFs) and extractable fractions (EFs). HPH = high-pressure homogenization; CUF = chelator UF; lAUF = low-alkaline UF; AcUF = acid UF; AcUF—HPH—AcUF = acid UF of the AcUF subjected to HPH; WEF = hot water EF; CEF = chelator EF; lAEF = low-alkaline EF; AcUF—HPH—WEF = water EF of the AcUF subjected to HPH; AcUF—HPH—AcEF = acid EF of the AcUF subjected to HPH.

As a first step, the AIR was prepared from the blanched tomato tissue. Two different pectin extraction approaches were performed on the AIR as schematically shown in Figure 1. On the one hand, a stepwise approach of three extractions was implemented aiming to extract different pectin fractions. On the other hand, single-step extraction of pectin under acid conditions was performed. In addition to this, the possibility of HPH to facilitate further pectin extraction was assessed by performing water extraction followed by acid extraction on the original acid-unextractable fraction after being subjected to HPH. All extractions were performed on a medium scale using a temperature-controlled extraction set-up with continuous stirring.

#### 2.3.1. Generation of the Alcohol-Insoluble Residue

After thawing, the blanched tomato slices were mixed in a laboratory blender (Waring Commercial, Torrington, CT, USA) two times for three seconds and subsequently filtered over a 1-mm sieve in order to remove peels and seeds [44]. The AIR was prepared based on the method by McFeeters and Armstrong [45]. In short, 192 mL technical ethanol was added to 60 g sieved tomato tissue and mixed (Mixer B-400, Büchi, Flawil, Switzerland) three times for six seconds. The precipitated material was recovered by vacuum filtration using filter paper (MN615, Macherey-Nagel, Düren, Germany). Two precipitates were combined, re-dispersed in 192 mL technical ethanol by mixing (Mixer B-400, Büchi, Flawil, Switzerland) three times for six seconds. The precipitate, again recovered by vacuum filtration, was dispersed for 10 min in 192 mL technical acetone by stirring. Next, the acetone was removed by vacuum filtration and the precipitate was dried overnight at 40 °C to obtain dry AIR. After grounding with mortar and pestle, the AIR was kept in a well-closed container until further use.

#### 2.3.2. Stepwise Pectin Extraction

The extraction conditions of the stepwise extraction approach were based on the procedures of Houben et al. [30] and Renard and Ginies [34]. Generally, after each extraction step, the extractable fraction (EF) and the unextractable fraction (UF) were separated by centrifugation (4–16KS, Sigma, Osterode am Harz, Germany) at 8000 g for 10 min at room temperature. The supernatant was vacuum-filtered (MN615, Macherey-Nagel, Düren, Germany) to obtain the respective EF and was stored at −40 °C until further analysis after being adjusted to pH 6 with 10 M HCl or 10 M NaOH (depending on the initial pH). If generation of a particular UF was only an intermediate step in the extraction sequence, the pellet and the very limited amount of precipitate on the filter were dispersed in the extraction medium of the next step. On the other hand, if generation of a particular UF constituted the aim and the stepwise extraction procedure was thus finished, the pellet was rinsed by dispersing in 3 L deionized water for 10 min. This mixture was centrifuged again under the same conditions. After decanting the supernatant, the respective UF was obtained as the pellet of the second centrifugation step. The UFs were vacuum-packed in plastic bags, frozen with liquid N_2_ and stored at −40 °C until further analysis. The different CWM fractions generated by the stepwise extraction are shown in Figure 1. All stepwise extractions were performed in duplicate.

##### Extraction with Hot Water

Deionized water at pH 4 (adjusted with HCl) was heated. Upon reaching 100 °C, 60 g AIR was added to the water and continuously stirred for 5 min. Next, the extraction mixture was cooled down to room temperature in an ice bath. The hot water-extractable fraction (WEF) was obtained by centrifugation and filtering as discussed above. The hot water-unextractable fraction was not studied as such and was always used for subsequent extraction(s).

##### Extraction with a Chelating Compound

The pellet obtained after extraction with hot water was dispersed in 4 L of 0.05 M CDTA in 0.1 M potassium acetate at pH 5. After 16 h of extraction at 28 °C under continuous stirring, the chelator-extractable fraction (CEF) and the chelator-unextractable fraction (CUF) or pellet for the next extraction step was obtained as discussed above.

##### Low-Alkaline Extraction

The pellet obtained after the extraction with CDTA was dispersed in 4 L of 0.05 M Na_2_CO_3_ solution. The extraction was performed for 22 h at 28 °C under continuous stirring. After the extraction, the low-alkaline-extractable fraction (lAEF) and the low-alkaline-unextractable fraction (lAUF) were acquired as discussed earlier.

#### 2.3.3. Acid Extraction and Acid Extraction Facilitated by High-Pressure Homogenization

##### Acid Extraction

The acid extraction was performed as described by Willemsen et al. [46]. First, 60 g of AIR was dispersed in 4 L of deionized water at 80 °C for 30 min under continuous stirring. After 30 min of incubation, the pH of the mixture was adjusted to 1.6 using 7 M HNO_3_ followed by 1 h of extraction while being stirred. The dispersion was cooled down in an ice bath to room temperature. The acid-extractable fraction (AcEF) and the acid-unextractable fraction (AcUF) were obtained (Figure 1) and stored in the same way as the EFs and UFs obtained by the stepwise extraction (Section 2.3.2). The acid extraction was performed in duplicate.

##### High-Pressure Homogenization of the AcUF

HPH was performed on a suspension of the AcUF [46]. For this, the AcUF was thawed and a 2% (*w*/*w*) suspension in deionized water was prepared based on the dry matter content of the AcUF and adjusted to pH 4.5 with 2 M Na_2_CO_3_. The suspension was kept overnight to assure complete hydration of the CWM. The dry matter content of the AcUF was determined according to Bernaerts et al. [47] with slight modifications. Briefly, 2 g AcUF was dried at 70 °C in a vacuum oven (UniEquip, Planegg, Germany) at a stepwise decrease in pressure level: 1 h at 0.8 bar, 1 h at 0.6 bar, 1.5 h at 0.4 bar and 0.5 h at 0.2 bar. Based on the amount of dry material and the initial amount of wet AcUF, the dry matter content was calculated. The suspension was mixed for 10 min at 8000 rpm (Ultra Turrax T25, IKA, Staufen, Germany) and afterwards high-pressure-homogenized at 20 MPa (Panda 2k, GEA Niro Soavi, Parma, Italy). The chosen pressure level was deemed relevant in the context of food industry and was expected to result in changes in pectin extractability because of the applied physical disruption [36,39]. The HPH treatment was performed in duplicate.

##### Water and Acid Extraction on the High-Pressure-Homogenized AcUF

The high-pressure-homogenized suspension was centrifuged at 8000 g for 10 min (4-16KS, Sigma, Osterode am Harz, Germany). The supernatant was filtered to obtain the water-extractable fraction of the AcUF subjected to HPH (AcUF—HPH—WEF) containing the CWM which was released from the cell wall matrix of the AcUF into the serum phase by HPH. The second nitric acid extraction was performed on the pellet, which was dispersed in deionized water using the same ratio of added water to the CWM as in the first acid extraction. The extraction process and the subsequent steps were the same as performed during the first acid extraction. The acid-extractable and the acid-unextractable fractions of the AcUF subjected to HPH are respectively denoted as AcUF—HPH—AcEF and AcUF—HPH—AcUF (Figure 1). These extractions were performed in duplicate.

#### 2.3.4. Lyophilization of the Extractable and Unextractable Fractions

The EFs and UFs were lyophilized before further characterization (Alpha 2–4 LSC plus, Christ, Osterode am Harz, Germany). In the case of the EFs, the respective liquid extracts were transferred into dialysis tubes (Spectra/Por^®^ Dialysis membrane, molecular weight cut-off: 3.5 kDa) and dialyzed extensively for 48 h against deionized water, which was replaced regularly by fresh deionized water, aiming to remove most of the salts, single monosaccharides and oligomers with a molecular mass (MM) lower than 3.5 kDa present in the extracts. The UFs were not dialyzed as they were already rinsed after the extraction process as discussed above. The freeze-dried CWM fractions were kept in a desiccator until further use.

### 2.4. Characterization of the Alcohol-Insoluble Residue and Cell Wall Material Fractions

#### 2.4.1. Analysis of the Uronic Acid Content

The uronic acid (UA) content of the AIR, UFs and EFs was determined by hydrolysis of the CWM based on the method of Ahmed and Labavitch [48] followed by determination of the UA content of the hydrolysates by the spectrophotometric method of Blumenkrantz and Asboe-Hansen [49]. Briefly, 10 mg of the AIR, UF or EF was hydrated overnight in 2 mL deionized water. The sample was hydrolyzed in duplo by adding 8 mL of concentrated sulfuric acid (95%) while being stirred in an ice bath. After 5 min, 2 mL deionized water was added dropwise to the mixture. The hydrolysis continued for 1 h and was stopped by diluting the hydrolysate to 50 mL in the case of the AIR, UFs and CEF or to 100 mL for the other EFs. To determine the UA content of the diluted hydrolysate, 3.6 mL sulfuric acid with sodium tetraborate (0.0125 M) was added to 0.6 mL diluted hydrolysate and heated at 100 °C for 5 min. After cooling, 60 µL of 0.15% (*w/v*) m-hydroxydiphenyl in 0.5% (*w/v*) NaOH was added to the sample and mixed for 1 min. For each hydrolysate, a blank was included by adding 0.5% (*w/v*) NaOH instead of 0.15% m-hydroxydiphenyl. Based on the resulting absorbance after 1 min at 520 nm (spectrophotometer Genesys 30 Vis, Thermo Fisher, Waltham, MA, USA), the UA content was calculated using external standards of GalA.

#### 2.4.2. Analysis of the Neutral Monosaccharide Composition

High-performance anion exchange chromatography with pulsed amperometric detection (HPAEC–PAD) was used to analyze the neutral monosaccharide composition of the CWM fractions. As the first step, the CWM was hydrolyzed based on the procedure of Yeats et al. [50] following different hydrolysis procedures depending on the nature of the sample. In order to quantify all neutral monosaccharides present in the AIR and UFs, Saeman hydrolysis was performed on these CWM fractions. First, 150 µL of 72% (*w*/*w*) H_2_SO_4_ was added to 3 mg of the AIR or UF and vortexed. For 1 h, the sample was kept at 30 °C and vortexed every 10 min. Thereafter, 4200 µL ultrapure water was added to obtain 4% (*w/v*) H_2_SO_4_ and hydrolyzed for 1 h at 121 °C. For the EFs, hydrolysis without preceding swelling of the cellulose was performed. Briefly, 3 mg EF was hydrated overnight in 4200 µL ultrapure water. After addition of 150 µL of 72% (*w*/*w*) H_2_SO_4_ to reach a concentration of 4% (*w/v*) H_2_SO_4_ and vortexing, the sample was hydrolyzed for 1 h at 121 °C. All the hydrolysates were cooled down in an ice bath, neutralized with NaOH (50% *w*/*w*) and adjusted to 10 mL with ultrapure water. Although full hydrolysis of the CWM under these conditions was reached, the hydrolysates were centrifuged for 15 min at 20,000 g (4–16KS, Sigma, Osterode am Harz, Germany) as a precaution step to protect the column from any particles. The supernatant was kept at –40 °C until further analysis. For each sample, these steps were performed in duplicate.

The hydrolysates were analyzed by HPAEC–PAD to determine the neutral monosaccharide composition following the methods of Houben et al. [30] and Yeats et al. [50] with some modifications. The analysis was performed with a Dionex system (ICS-6000, Sunnyvale, CA, USA) consisting of a CarboPac^TM^ PA20 guard column, a CarboPac^TM^ PA20 analytical column and an ED50 electrochemical detector (Dionex, Sunnyvale, CA, USA). This detector operated in the PAD mode with a quadruple potential waveform as described by Houben et al. [30] and consisted of an Ag/AgCl reference pH electrode and a gold electrode [51]. The hydrolysates and, if needed, dilutions thereof were filtered (0.20 µm pore size, Chromafil, Macherey-Nagel, Düren, Germany) before injection. Two elution profiles were combined to achieve good separation of all the neutral monosaccharides. Elution with 18 mM NaOH (flow: 0.4 mL/min) was used to separate Rha and Ara, whereas the other monosaccharides, namely, fucose (Fuc), Gal, Glc, Xyl and mannose (Man), were separated using 2 mM NaOH elution (flow: 0.4 mL/min). Briefly, the column was equilibrated for 10 min at 2 or 18 mM NaOH followed by injection of 10 µL of the (diluted) hydrolysates. Next, isocratic elution was maintained for 25 min for 2 mM NaOH elution and 15 min in the case of 18 mM NaOH elution, both at 30 °C. Finally, after each run, the column was regenerated by elution with 500 mM NaOH for 10 min. Identification and quantification of the analyzed monosaccharides was achieved by a mixture of monosaccharide standards (L-Fuc, L-Rha, L-Ara, D-Gal, D-Glc, D-Xyl and D-Man) at different concentrations (1–6 ppm) as the external standard. In order to correct for the partial degradation of the monosaccharides during hydrolysis with 4% (*w/v*) H_2_SO_4_, recovery values were calculated by exposing the sugar standard mixture with a known concentration to hydrolysis at 121 °C with 4% (*w/v*) H_2_SO_4_.

#### 2.4.3. Analysis of the Degree of Methyl Esterification

The DM of the pectin in the WEF, AcEF, AcUF—HPH—WEF and AcUF—HPH—AcEF was determined by Fourier-transform infrared (FT-IR) spectroscopy [52]. Lyophilized EF was compacted on the sample holder of a FT-IR spectrophotometer (IRAffinity-1, Shimadzu, Tokyo, Japan). The FT-IR spectrum was deconvoluted in order to avoid interference by proteins present in the CWM fraction. Based on the intensity of absorption at wavelengths around 1600 (COO– stretch) and 1740 cm^−1^ (C=O stretch), the DM was estimated according to the calibration curve developed by Kyomugasho et al. [52]. For each sample, the analysis was performed in duplo.

#### 2.4.4. Analysis of Molecular Mass Distribution of the Extractable Fractions

Of each EF, 40 to 100 mg was solubilized overnight in 5 mL ultrapure water. Further preparation of the sample was based on the procedures of Kaya et al. [53]. The solution’s pH was adjusted to 2 using 1 M HNO_3_, after which 15 mL pure isopropanol was added. The EF was kept overnight in this 75% (*v/v*) isopropanol solution at 4 °C. After centrifugation at 40,000× *g* for 15 min at 4 °C (Avanti JXN-26, Beckman Coulter, Indianapolis, IN, USA), the supernatant was decanted and the precipitate was dispersed again in 20 mL of 75% (*v/v*) isopropanol by 5 min of intensive shaking. In order to recover the precipitate, centrifugation at 40,000× *g* for 15 min at 4 °C was performed again. After decanting the supernatant, the precipitate was dried at room temperature for at least 48 h. In order to prepare the samples for analysis, 10 mg of this dried material was solubilized overnight in 3.5 mL ultrapure water. Next, the solution was heated for 10 min at 50 °C, cooled down to room temperature, adjusted to pH 6 with 0.05 M LiOH or 0.5 M LiOH in the case of the CEF and lAEF and diluted to 5 mL with ultrapure water. The 0.2% (*w/v*) solution was heated again at 50 °C for 10 min and immediately filtered through a filter (0.45 µm pore size, Chromafil, Macherey-Nagel, Düren, Germany).

The MM distribution of the EFs was analyzed by size exclusion chromatography coupled with multiangle laser light scattering (PN3621, Postnova Analytics, Landsberg am Lech, Germany) and a refractive index detector (Shodex RI-101, Showa Denko K.K., Kawazaki, Japan) as described by Shpigelman et al. [54] with minor changes. Briefly, 100 µL filtered 0.2% (*w/v*) solution of each EF prepared as discussed above was injected by an autosampler (G1329A, Agilent Technologies, Diegem, Belgium). The sample was eluted with 0.1 M NaNO_3_ in a 0.1 M acetic acid buffer (pH 4.4) at a flow rate of 0.5 mL/min over three Waters columns in series (Waters, Milford, MA, USA), namely, Ultrahydrogel 250, 1000 and 2000, with exclusion limits 8 × 10^4^, 4 × 10^6^ and 1 × 10^7^ Da, respectively, which were kept at 35 °C. Based on the dn/dc value of 0.146 mL/g, the concentration was obtained, whereas the MM was calculated using the Debye fitting method (up to the 2nd order) of the software (NovaMals, version 1.2.0.0, Postnova Analytics, Landsberg am Lech, Germany). For each sample, the analysis was performed in duplicate.

### 2.5. Statistical Analysis

Since all the extractions were performed in duplicate, each EF and UF was prepared twice. For the big AIR batch, two samples were taken prepared on different days. On both duplicates of the AIR and each EF and UF, each analysis was performed twice, indicated as n = 2.2. The data are reported as the average of these four measurements ± standard deviation.

## 3. Results

### 3.1. Uronic Acid Extraction Efficiency of the Different Pectin Extraction Procedures

**Figure 2:**
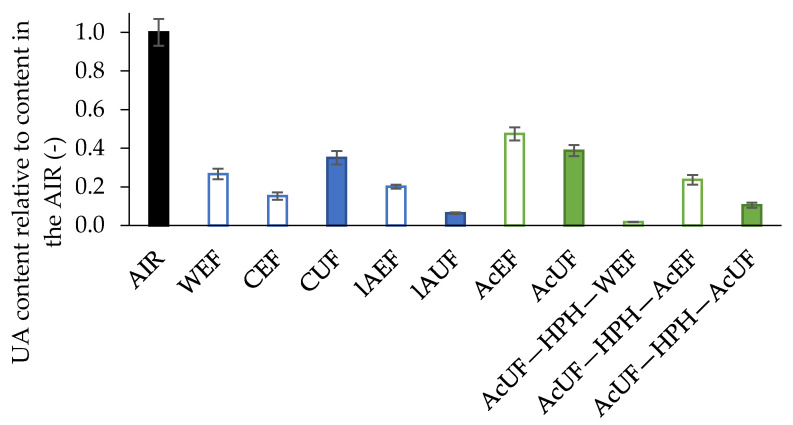
Uronic acid (UA) content relative to the content in the alcohol-insoluble residue (AIR) for the extractable fractions (EFs) and unextractable fractions (UFs). The error bars represent the standard deviation (n = 2.2).

Two pectin extraction approaches were compared in this study (Figure 1). On the one hand, pectin was extracted by the stepwise use of different pectin extraction media. On the other hand, single-step acid pectin extraction was performed, followed by water and acid extraction facilitated by HPH. In what follows, the pectin extraction yield for both methods is compared. The pectin extraction yield of the different extraction approaches can be addressed by comparing the UA content in the respective EFs as a measure of pectin. It should be noted that UA covers both the monosaccharides GalA and glucuronic acid (GlcA). As, in the case of tomato, GlcA is almost exclusively present in glucuronoxylan [40,55], the contribution of GlcA to the UA for the EFs is very low. For the AIR and UFs, however, part of the UA originates from GlcA, but is, based on Broxterman and Schols [40] and Reinders and Thier [55], assumed small in comparison to the amount of GalA originating from (residual) pectin present in these fractions. As GalA is by far the most predominant UA, UA was used to quantify GalA and, by extension, to evaluate the (residual) pectin content present in the CWM. In Figure 2, the UA content in the EFs and UFs expressed relative to the UA content in the AIR is presented, showing the UA extraction efficiency of the different methods as well as the percentage of UA retained in the CWM after extraction. Since the EFs were dialyzed in membranes with a MM cut-off of 3.5 kDa, the measured UA content was expected to originate almost exclusively from pectin polymers and not from single units or small oligosaccharides (<3.5 kDa) which were initially present or arose during extraction because of β-elimination (at high temperature and neutral pH) [26,29] or acid hydrolysis (at high temperature and low pH) [53,56]. The possible effect of these reactions during extraction are addressed further when discussing the structural properties of the extracted pectin.

The highest yield during the stepwise pectin approach was obtained by the first extraction step with hot water. Approximately 27 ± 3% of pectin (based on UA) of tomato was extracted as the WEF, containing pectin that was weakly interacting in the cell walls of processing tomato. Additional pectin was extracted with CDTA, which allows solubilizing calcium-bound pectin, and under low-alkaline conditions, solubilizing pectin which interacts in cell walls by low-alkali-labile bounds. The combined UA extraction yields of the first and second extraction and all extraction steps of the stepwise extraction were 42 ± 3% and 62 ± 4%, respectively. Although the former pectin extraction yield was in accordance with Broxterman and Schols [33], these authors observed that the highest yield was obtained during the extraction using a chelating agent instead of hot water. In contrast, the single-step acid extraction procedure resulted in a yield of 47 ± 3% (obtained as the AcEF). As expected, the amount of UA obtained by acid extraction was higher than for hot water extraction (5 min at 100 °C) due to low pH (1.6) in combination with high temperature (80 °C) and longer extraction time (60 min), allowing solubilization of more strongly interacting pectin. Improved extractability of pectin upon lower extraction pH was also shown by Kaya et al. [53] for citrus fruits and by Denman and Morris [56] for melon fruit. However, as also reported for citrus peel CWM by Willemsen et al. [46], single-step acid extraction on processing tomato CWM could not reach the same UA extraction efficiency as all the three steps of the chemical stepwise method combined. This can also be observed by the higher retention of UA in the AcUF than in the lAUF (Figure 2).

Although a less complete pectin extraction could be achieved by the single-step acid extraction than for the stepwise approach, an important benefit of the single-step acid extraction is its convenience, especially in the context of time and number of handlings needed. In this context, based on the results shown in Figure 2, it is clear that more UA and thus pectin was lost when more extractions were performed in sequence. The total fraction of UA which was recovered in the different CWM fractions (EFs and the final UF) obtained after full stepwise extraction (i.e., WEF + CEF + lAEF + lAUF) was 68 ± 4%, whereas this value was 86 ± 4% in the case of single-step acid extraction (i.e., AcEF + AcUF). The former recovery was in good accordance with the results obtained by Houben et al. [30] who performed similar extractions on tomato. This loss of UA can be attributed to the increasing amount of intermediate handlings resulting in higher losses of material, which was also mentioned by others [30,46]. Next to that, the dialysis step and the washing step performed on the EFs and UFs, respectively, could contribute to the losses observed.

Especially in comparison to the small amount of UA left in the lAUF (6.4 ± 0.5% of the amount of UA in the AIR), a considerable of amount of UA was still retained in the AcUF (39 ± 3%). As the next step, it was addressed whether the extractability of pectin by water and acid could be improved by preceding mechanical disruption of the AcUF by HPH. After performing HPH on a 2% suspension of the AcUF, a small amount of the serum phase was obtained as a supernatant under the applied centrifugation conditions (AcUF—HPH—WEF), containing only 1.8 ± 0.2% of the total amount of UA present in the AIR. On the pellet, the second acid extraction was performed, which resulted in a UA extraction yield of 24 ± 3%. In total, 65 ± 8% of the UA present in the AcUF was obtained in the AcUF—HPH—WEF and AcUF—HPH—AcEF. In other words, almost two thirds of pectin which were not extractable during the single-step acid extraction was released into the serum phase (AcUF—HPH—WEF) or was extractable during repeated acid extraction (AcUF—HPH—AcEF) facilitated by the preceding HPH at 20 MPa for a single pass. It should be noted that part of the pectin in the serum phase is expected to be obtained as AcUF—HPH—AcEF instead of as AcUF—HPH—WEF because of the stability of the suspension towards the applied centrifugation conditions. 

The improving effect of HPH at 20 MPa on the extractability of pectin from cell walls of processing tomato could be attributed to the mechanically disruptive action of HPH which disturbed or weakened the interactions of pectin in the CWM contained in the AcUF. The effect of HPH on the extractability of tomato pectin was also studied by Christiaens et al. [39] who showed that part of the initially low-alkaline-extractable pectin shifted to chelator-extractable pectin upon HPH at 10 MPa of high-temperature-blanched tomato tissue. Moelants et al. [57] observed that HPH of blended tomato tissue after mild or strong heat treatment at 20 MPa and 100 MPa did not result in a significantly higher UA content in the serum phase than before HPH. However, it should be noted that in both studies, HPH was performed on mixed tomato tissue, whereas in our study, HPH was performed on tomato CWM which was already partially pectin-depleted. It is plausible that the preceding acid extraction contributed to the potential of HPH in improving the extractability of the residual pectin. Removing pectin from the cell wall could probably, as also suggested by Redgwell et al. [58] and Willemsen et al. [46], lead to interfibrillar spaces and voids within the hemicellulose and cellulose network. As discussed by Sankaran et al. [59], loosing interactions between cell wall polymers could induce a loss in strength of the cell wall, making the CWM more prone to rupture under shear force. As a consequence, the preceding pectin depletion step probably contributed to easier disruption and disturbance of the cell wall network by HPH, favoring the release and extraction of pectin from the cell wall by weakening the interactions and creation of a more open cell wall structure which is more accessible for the acid to solubilize pectin. 

Finally, 10 ± 1% of UA from the AIR was still retained in the AcUF—HPH—AcUF, whereas this value only amounted to 6.4 ± 0.5% in the case of the lAUF. Together with a small amount of glucuronoxylan, this residual UA content corresponded to a pectin fraction which was very strongly interacting in the cell wall, probably by interactions with hemicellulose and cellulose. To extract such a fraction, stronger alkaline conditions or more intensive disruptive techniques are needed. 

### 3.2. Chemical Properties of Extractable Fractions in Relation to the Extraction Method

#### 3.2.1. Monosaccharide Composition of the Extractable Fractions

The monosaccharide composition of the EFs can be found in Table 1 expressed as mg monosaccharide per g dry EF (mg/g). As expected, UA is the most prominent among the monosaccharides characterized, indicating that the main polysaccharide present in the EFs was pectin. Neckebroeck et al. [44] found a higher relative amount of Gal to Rha for nitric acid-extracted pectin, which was probably related to the different batch of tomato since the obtained Gal content for the AcEF was in line with the content of Gal obtained for the other EFs and also with the content in the AIR and the UFs (cfr. 3.3). As Fuc was not found in detectable amounts in any of the CWM fractions, this monosaccharide was not further considered in the discussion. However, it should be noted that the total amount of polysaccharides in the EFs was different. On the one hand, the total amount of polysaccharides (calculated as the sum of the monosaccharides determined) in the WEF, AcEF, AcUF—HPH—WEF and AcUF—HPH—AcEF ranged between 616.5 to 747.5 mg/g. These summed values are in accordance to what was obtained by Neckebroeck et al. [44] for acid-extracted tomato pectin and somewhat higher than those obtained by Guillon et al. [60] for water- and oxalate-extracted tomato pectin. Because of the relatively high protein content in the CWM of tomato [44,55] and the presence of salts, it is very reasonable that not all material in the EFs can be attributed to polysaccharides. The lower total monosaccharide content found for the lAEF and especially for the CEF originated from contamination with CDTA, which could not be completely avoided even by the extensive dialysis step performed before lyophilization.

To gain more understanding into the polysaccharide composition and some relevant structural properties of the EFs, the contribution of HG (mol%) and RG-I (mol%) and some relevant molar ratios were calculated based on the monosaccharide content and are presented in Table 2. In accordance with Denman and Morris [56], the purity of the pectin extract is evaluated by the ratio of the sum of the typical pectin monosaccharides (UA, Rha, Ara and Gal) to the sum of the monosaccharides originating from other polysaccharides (Glc and Man) (Table 2). In the EFs, it is not straightforward to allocate Xyl to pectin or co-extractants as Xyl is a constituent of the xylogalacturonan region of pectin [7], but is also one of the main monosaccharide in xyloglucan [61]. In the case of tomato, part of this latter polysaccharide has been shown to be water-extractable [62]. For this reason, it was decided not to take the amount of Xyl into account to calculate pectin purity of the EFs. A high value for the ratio (UA + Rha + Ara + Gal)/(Glc + Man) is thus indicative of a low amount of co-extracted non-pectic polysaccharides (Table 2).

The lowest values, although they still indicate high purity, were obtained for the WEF and CEF, whereas the values were very high for the lAEF, AcUF—HPH—WEF and AcUF—HPH—AcEF. The value for the AcEF was in between the values obtained for the EFs of the chemical stepwise approach. In the WEF, CEF and AcEF, a small amount of Glc was found, which could be attributed to a small amount of co-extracted starch during the first step(s) of the extraction procedures [44,60]. Since the amount of starch in tomato decreases during ripening [60], the low value of Glc in these extracts is reasonable as tomatoes were used in their red-ripe stage. In addition, part of the Glc present in the EFs could be attributed to xyloglucan. Especially in the case of the AcUF—HPH—WEF and AcUF—HPH—AcEF, it is probable that most of the Glc can be attributed to xyloglucan, taking into account the extraction of starch during the first acid extraction step and the concomitant presence of Xyl. Next to the presence of Glc, the lower value for the pectin extract purity in the WEF and CEF was also partly attributed to the presence of Man, which was also found by Houben et al. [30] for water-extracted pectin from tomato. Man was also encountered by Reinders and Thier [55] in material extracted with water and ethylenediaminetetraacetic acid (EDTA) from the alcohol-insoluble solids of tomato, which could suggest a small amount of (hot) water-extractable mannans. The applied shear to the AcUF could probably improve water and acid extractability of a specific fraction of hemicellulose. However, it should be emphasized that the amount of hemicellulose in the EFs is very small and, in practice, negligible. 

The total proportion of HG (mol%) and RG-I (mol%) was calculated as UA–Rha and 2Rha + Gal + Ara, respectively [56] and is shown in Table 2. The proportion of HG was around 85 mol% for all the EFs, whereas the abundance of RG-I, taking both backbone and side chains into account, ranged between 9.9 mol% (for the CEF) and 14.3 mol% (for the lAEF). More information on the structural properties of the pectin extracted under different conditions can be obtained by comparing the relative abundances of the main pectin monosaccharides (UA, Rha, Ara and Gal) and its ratios. Although the variability in proportion of RG-I between the EFs was only moderate, the properties of these RG-I domains were clearly distinct. In order to facilitate understanding of these RG-I structural properties, two ratios defined by Houben et al. [30], namely, Rha/UA and (Ara + Gal)/Rha, were compared between the EFs (Table 2). When the ratio Rha/UA, which is a measure of the contribution of the RG-I domains to the pectin backbone, is higher, more and/or longer RG-I domains are present in the pectin backbone of an EF, whereas the ratio (Ara + Gal)/Rha gives an indication of the extent of branching of RG-I. When this ratio is higher, the RG-I domains have more and/or longer side chains. When both values are higher, of course, the contribution of RG-I as a whole (backbone and side chains) to the pectin in the respective EF is higher. 

First of all, it is clear that less harsh extraction conditions, namely, hot water and with CDTA, resulted in extracted pectin which was characterized by smaller or a lower amount of RG-I domains in the pectin backbone than pectin which was extracted under harsher conditions, concerning both harsher chemical conditions as applied during the acid and low-alkaline extraction as well as physically disruptive forces in the case of HPH of the AcUF enabling improved subsequent water and acid extraction. Following the extraction design, it should be mentioned that the higher value for Rha/UA for harsher extraction conditions is partly attributed to the preceding extraction of pectin which is poorer in RG-I backbone, e.g., when the extraction under low-alkaline conditions would be performed on the AIR instead of on the CUF, the ratio Rha/UA is expected to be lower. A higher Rha/UA ratio in the case of the lAEF than for the WEF and CEF of tomato was also found by Houben et al. [30]. Broxterman and Schols [40] also reported that solubilization of HG-rich pectin from the CWM matrix is easier than of RG-I-rich pectin, as the RG-I/HG ratio of water-extracted pectin from planetary ball-milled chelating agent-unextractable solids of tomato increased with milling time.

The extent of RG-I branching, as quantified by the ratio (Ara+Gal)/Rha in Table 2 was highest for pectin extracted with water (WEF) and slightly lower for pectin extracted with a chelator (CEF). The conditions during extraction with hot water and the subsequent extraction with CDTA generally only result in the extraction of weakly bound pectin and pectin interacting by divalent cations, respectively [34]. In the case of these latter pectin–pectin interactions, a higher extent of branching could even reduce the interaction strength [30,63], rationalizing the higher (Ara + Gal)/Rha ratio found for the WEF than for the CEF. In the case of processing tomato, the RG-I domains of pectin which were extracted with 0.05 M Na_2_CO_3_ favoring the release of ester-bound pectin [34] seemed to be characterized by an even lower extent of branching. The trend between the extent of branching and extraction conditions was in accordance with the observations by Houben et al. [30].

However, in the context of extraction of pectin under acidic conditions, it has to be stipulated that the extent of branching of RG-I is not only affected by the chemical properties of the pectin population extracted under these specific conditions, but also by the hydrolytic action of the acid. Indeed, the effect of the acid during extraction is twofold; it results not only in the solubilization of pectin, but also in the hydrolysis of pectin to which especially the neutral sugar side chains are prone due to acid lability of its linkages [25,53,64], which rationalizes the lower ratio of (Ara + Gal)/Rha on the one hand for the AcEF than for the WEF and CEF and, on the other hand, for the AcUF—HPH—WEF and AcUF—HPH—AcEF than for the lAEF. 

The acidic environment during the second acid extraction step could also contribute to a lower value of the (Ara + Gal)/Rha ratio. However, this effect seems to be limited as the ratio was equal for the AcUF—HPH—WEF and AcUF—HPH—AcEF. Based on the ratio (Ara + Gal)/Rha, which was lower than 1 for the AcUF—HPH—WEF, the side chains which were present in the extractable pectin at the start of the second acid extraction are expected to be very short or even consisting of one monosaccharide unit, leading to very few linkages in the side chains which could be hydrolyzed. As a consequence, the effect of the low pH during the second extraction on the (Ara + Gal)/Rha ratio was less outspoken than during the first extraction. The lower value for (Ara + Gal)/Rha found for pectin obtained after subjecting the AcUF to HPH in comparison to the AcEF is, as a consequence, most probably attributed to the intrinsic chemical properties of the more strongly interacting pectin fraction released by the high shear forces exerted by HPH. This observation is in accordance with the pectin fraction obtained by low-alkaline extraction as the last step of the chemical stepwise extraction approach. Since the compositional and structural properties of the AcUF—HPH—WEF and AcUF—HPH—AcEF based on the monosaccharide composition are very similar, it is suggested that by HPH, a specific, more strongly interacting pectin fraction which is not extractable by single-step acid extraction is partially released to the serum phase (AcUF—HPH—WEF) and, furthermore, made available by HPH for repeated acid extraction (AcUF—HPH—AcEF). Based on the combined insights of the two pectin extraction approaches aiming at extensive pectin extraction, it can be suggested that more strongly interacting tomato pectin is characterized by a higher RG-I content and a lower extent of branching. However, this is true for the pectin which was extractable, as the structural properties of the pectin which was unextractable under the presented pectin extraction approaches (Figure 1) and thus strongly bound to the cell wall, e.g., by interaction with hemicellulose and cellulose, were not further considered.

#### 3.2.2. Molecular Mass of the Extractable Fractions

**Figure 3:**
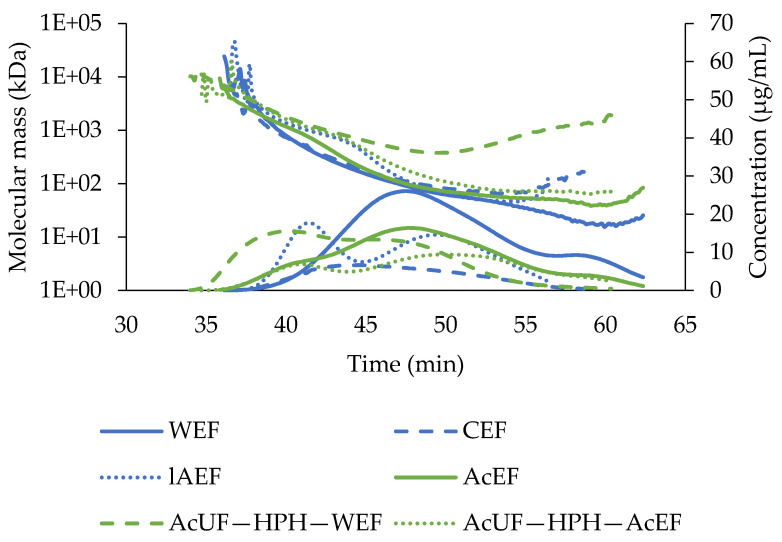
Molecular mass distribution of the extractable fractions (EFs). The curves represent the elution profile and the slanting lines on top show the molecular mass of the polymers eluting.

For each EF, the MM distribution obtained by size-exclusion chromatography is presented in Figure 3. The elution profile of the pectin molecules of the WEF was monomodal and covered mainly low MM pectin. Both the CEF and lAEF consisted of small and large pectin molecules. More specifically, as shown in Figure 3, the MM distribution of the CEF was monomodal but broad, whereas for the lAEF, a more bimodal distribution was observed. By overlaying the distributions of the WEF, CEF and lAEF, one can conclude that the overall MM distribution of the extracted pectin from tomato is relatively broad and rather monomodal.

Based on the average MM (Table 1) of the different EFs of the chemical stepwise approach, the highest value was obtained for the lAEF (451.0 kDa), whereas the lowest value was obtained for the WEF (114.8 kDa), while it was intermediate for the CEF (233.0 kDa). It thus seemed that the average MM increased with the capability of the extraction medium to break interactions. As high temperature was applied to the material during water extraction, β-elimination could occur, having a negative impact on the pectin’s MM [26]; however, the use of hot water instead of cold water could also improve the extraction of pectin with a higher MM [29]. Although in the case of the WEF a small percentage of large molecules appeared, the average MM of hot water-extractable pectin was much lower than the peak in MM around 404–778 kDa observed for hot water-extractable pectin of tomato by Houben et al. [30]. However, Lin et al. [65] observed that the average MM of different fractions of water-soluble pectin obtained from tomato pastes (both hot and cold break) ranged between 9 and 162 kDa and was more in accordance with the value reported in the current study.

A broad MM distribution was observed for the AcEF, not only covering the MM region of pectins in the WEF, but also including a tail at a higher MM at similar elution times like the first peak of the lAEF. The presence of a (small) fraction of large pectin molecules in the AcEF is in line with the results of the monosaccharide compositions of the EFs. Indeed, the Rha/UA ratio of the AcEF was in between the value for the WEF and CEF on the one hand and for the lAEF on the other hand, which also showed that acid conditions enabled extraction of more strongly interacting pectin together with more loosely bound pectin. The average MM determined by Neckebroeck et al. [44] of tomato pectin extracted under acid conditions using the same conditions as in our study was around 523 kDa, which was almost double the result for the AcEF (270.3 kDa) in Table 1. This discrepancy could be attributed to a different batch of tomatoes used.

The average MM of the pectin molecules in the AcUF—HPH—WEF was high (1507.5 kDa) in comparison to the molecules in the lAEF. This observation suggests that high shear forces exerted during HPH on the AcUF allowed releasing pectin in a more intact way than by extraction under low-alkaline conditions whereby the pectin was probably solubilized by limited cleavage of covalent bonds (e.g., ester bonds). It was shown for citrus pectin, which is also rather linear, that HPH only had a significant decreasing effect on the MM of pectin polymers at pressure levels above 150 MPa [66]. As the applied pressure in our study was only 20 MPa and the MM of the AcUF—HPH—WEF was high, it is expected that the pectin interactions in the cell wall were weakened without breaking covalent bonds in the pectin chain. The average MM and MM distribution of the pectin molecules extracted under acid conditions after HPH, i.e., the AcUF—HPH—AcEF (509.5 kDa), and low-alkaline conditions, i.e., the lAEF (451.0 kDa), were rather similar. On the other hand, the outspoken difference in the average MM of the AcUF—HPH—WEF and the AcUF—HPH—AcEF could be related to the acid environment during acid extraction after HPH. Especially in the case of the pectin molecules which were released from cell walls only by physical disruption and characterized by a high MM, it is reasonable that even very few hydrolytic cleavages per chain resulted in a tremendous decrease in MM, leading to the very broad MM distribution (Figure 3), which was found for the AcUF—HPH—AcEF. Since it has been reported that a higher MM improves the strength and stiffness of pectin gels [67,68], the EFs with higher MM pectin are possibly more appropriate to be used as a gelling agent. 

#### 3.2.3. Degree of Methyl Esterification of the WEF, AcEF, AcUF—HPH—WEF and AcUF—HPH—AcEF

The DM was determined for the WEF, AcEF, AcUF—HPH—WEF and AcUF—HPH—AcEF and is shown in Table 1. DM measurement was not performed on the CEF and lAEF for two reasons. On the one hand, the residual amount of CDTA present in the CEF and lAEF would interfere with the FT-IR method for determination of the DM. On the other hand, as saponification occurs under low-alkaline conditions [69], a very low DM would be obtained for the lAEF which is not representative for the DM of this pectin fraction as it occurs in tomato tissue. The highest DM was found for the WEF (73.8%). As a consequence of the high DM, the interaction between HG chains mediated by calcium crosslinks is not favored. Together with the low amount of RG-I domains and the relatively high extent of branching, it is clear that the pectin in the WEF is the less strongly bound pectin. Houben et al. [30] obtained an even higher DM for water-extractable pectin of tomato (91.3%). A slightly lower DM was found for the AcEF (66.0%) than for the WEF. In the case of tomato, this DM is greatly dependent on the ripeness of the tomato [70]. The reported DM for acid-extracted pectin by Neckebroeck et al. [44] was 54.3% and thus comparable to the value obtained for the AcEF. In the current study, red-ripe tomatoes which were not yet clearly softening were studied and immediately high-temperature-blanched to avoid any further structural changes of the pectin by enzymes, e.g., demethoxylation by pectin methylesterase. The DM of the pectin in the AcUF—HPH—WEF (60.5%) was slightly lower than the DM obtained for the AcEF (66.0%), whereas the DM of the AcUF—HPH—AcEF was remarkably low (47.0%). This lower DM for the AcUF—HPH—AcEF was probably the result of two factors. On the one hand, it seems that more strongly interacting pectin was characterized by a slightly lower DM, as shown by the difference between the AcEF and AcUF—HPH—WEF. In this context, pectin which was not yet readily available after HPH, thus present in the AcUF—HPH—AcEF, could have an even lower DM than the AcUF—HPH—WEF. On the other hand, low pH (pH = 1.6) during extraction could have resulted in some acid pectin demethoxylation and thus in a slightly lower DM [56,71].

### 3.3. Monosaccharide Composition of the Alcohol-Insoluble Residue and Unextractable Fractions

The monosaccharide content (mg/g) of the AIR and the different UFs is shown in Table 3. The most abundant monosaccharide in the AIR and the UFs is Glc, originating from cellulose, some hemicelluloses and starch. The contribution of the latter is expected to be very low as discussed earlier. Since the content of Glc and the typical hemicellulose monosaccharides, Xyl and Man, clearly increased from the AIR over the CUF/AcUF to the lAUF/AcUF—HPH—AcUF, it is confirmed that, as expected, the relative cellulose and hemicellulose content increased upon pectin depletion. The high pectin extraction yield obtained after the complete stepwise extraction and the repeated acid extraction with intermediate HPH (cfr. 3.1) was also reflected in the very low content of UA and Rha in the lAUF and AcUF—HPH—AcUF.

A clearly lower Ara content was observed for the AcUF and AcUF—HPH—AcUF due to hydrolysis when exposed to the acidic environment during extraction, to which especially arabinan side chains are susceptible [22]. Apart from that, the content of Ara and Gal was similar for the AIR and the different UFs independent of the residual amount of pectin as estimated by UA. Although a decrease in the Ara and Gal content might be expected due to the decrease in the pectin content, this independency could be understood as follows. Upon extracting extensive amounts of pectin, the contribution of the strongly interacting pectin increases. Based on the recent findings showing that strong interactions between pectin and cellulose and hemicellulose were mainly exhibited by the neutral arabinan and galactan pectin side chains rather than the HG and RG-I backbone [15,72,73,74], the residual pectin could be expected to be rather Ara- and Gal-rich. Furthermore, the rather constant content of Gal, which is also a constituent of galactomannans, could partly be attributed to the higher contribution of these galactomannans in more pectin-depleted UFs as noticed by the increasing Man content. The presence of galactomannans in tomato CWM has also been shown by Reinders and Thier [55]. 

The monosaccharide composition of the AIR of tomato expressed in mol% is comparable to what was obtained by Broxterman and Schols [33] except for the amount of Ara and Gal which was clearly lower in our study, probably due to a different variety of tomatoes. Since the values are expressed relatively, slightly lower values for the mol% of UA and Glc were reported by these authors as well, namely, 31 and 42 mol% instead of 38 and 46 mol% in this research, respectively. The monosaccharide composition of the CUF was in accordance with the results of Broxterman and Schols [33] on the composition of chelating agent-unextractable solids of tomato CWM, for which Glc, Xyl, Man and UA accounted for 55, 8, 6 and 19 mol%, similar to the results of our study, namely, 58, 9, 6 and 22 mol%, respectively. Furthermore, the monosaccharide composition of the residual CWM from which pectin was extracted by a stepwise pectin extraction approach with water, EDTA and 0.1 M NaOH determined by the same researchers [33] resulted in very similar values (Glc, Xyl, Man and UA were respectively 69, 8, 6 and 8 mol%) to our stepwise approach leading to the lAUF (72, 11, 7 and 6 mol%, respectively). Based on this comparison, the efficiency to deplete the CWM of tomato in pectin by 0.05 M Na_2_CO_3_ or 0.1 M NaOH following a stepwise approach seemed very similar.

The monosaccharide composition of the UFs was used to gain more insight into the polysaccharide composition (mol%) and compositional molar ratios, which are presented in Table 4. The observation that the contribution of the pectin backbone (UA + Rha) (mol%) to the total polysaccharides in the AcUF—HPH—AcUF was clearly higher than in the lAUF suggests that in the case of processing tomato, consecutive extractions with conditions optimized to extract fractions of pectin with specific chemical properties is more efficient than the more general acid pectin extraction, even when the second acid extraction was performed assisted by HPH. Since the contribution of the pectin backbone in the CUF and AcUF was very similar, it is clear that the main factor determining the residual amount of pectin is the last step of both approaches. Based on this, one can conclude that low-alkaline conditions are more efficient in releasing pectin from the pectin-depleted cell wall network of processing tomato than acid extraction facilitated by high shear forces.

The main hemicelluloses in tomato are xyloglucan, galactomannan, glucomannan and glucuronoxylan [35,55]. The contribution of mannans to hemicellulose can be addressed by the ratio Man/Xyl [30]. This value (Table 4) was similar for all UFs and in good agreement with the value for the hemicellulose fraction of tomato obtained by Houben et al. [30]. Since this ratio showed no dependency on the extent of pectin depletion and only a small amount of Xyl is found in the EFs, which could also be originating from a very small amount of xyloglucans instead of xylogalacturonan as discussed above, it seems a justifiable approximation to attribute all Xyl in the AIR and UFs to the hemicellulose fraction together with all Man. 

The Glc content observed in the AIR and UFs originates from cellulose as well as from the hemicellulose fraction, of which xyloglucan is the most prominent as it is the main (Glc- and Xyl-containing) hemicellulose in cell walls of edible fruits of dicotyls. In the case of solanaceous plants such as tomato, xyloglucan has a substitution pattern of XXGG, meaning that two xylose units occur per four Glc units [5,35,61]. Given these considerations, the ratio of typical hemicellulose monosaccharides (Xyl and Man) to Glc was calculated to evaluate the relative changes between the hemicellulose and cellulose content. Although this value could be slightly biased for the AIR due to the small amount of starch present, the values for the AIR and UFs were all around 0.25, which confirms again that no significant amount of hemicellulose was co-extracted by the different pectin extraction methods studied. 

Finally, the total amount of hemicellulose and cellulose in the AIR and UFs was calculated based on the sum of the amount of Glc, Xyl and Man. It should be noted that this is an estimation, since, on the one hand, only the main hemicellulose monosaccharides were allocated to this fraction and, on the other hand, the amount of Glc and Xyl, respectively, originating from starch and xylogalacturonan was neglected. Nevertheless, based on what was discussed above, this allocation seems a valid assumption for the CWM of processing tomato. This value together with the contribution of the pectin backbone (mol%) facilitates an easy and general comparison on the composition which can be expected for processing tomato CWM fractions, which are obtained as residue after specific pectin extractions. The contribution of hemicellulose and cellulose was around 56 mol% for the AIR. As a result of pectin depletion, the contribution of hemicellulose and cellulose to the polysaccharides present in the cell wall was increasing. Indeed, when around half of the pectin content was extracted from the CWM as is the case for the CUF and AcUF, the contribution increased to around 73 mol%, whereas the full chemical stepwise approach or acid extraction followed by water and acid extraction facilitated by HPH resulted in almost 90 mol% of the polysaccharides being hemicellulose or cellulose. 

Apart from the differences in the contribution of the pectin backbone (mol%) for the lAUF and AcUF—HPH—AcUF (and the Ara content), it should be noted that the monosaccharide composition of the CUF and AcUF on the one hand and the lAUF and AcUF—HPH—AcUF were very similar as can be easily deduced from Table 4. Hence, the microstructural changes caused by the HPH treatment did not lead to significant extraction of hemicellulose during the subsequent acid extraction, which was also confirmed by the very small amount of Xyl and Man present in the AcUF—HPH—WEF and AcUF—HPH—AcEF.

## 4. Conclusions

In the current study, two different extensive pectin extraction approaches performed on the AIR of processing tomato were compared. Apart from the pectin extraction yield, the main emphasis of this study was on the characterization of the chemical structural properties of the extracted pectin and the composition of the residual unextractable CWM. The UA + Rha content in the CUF, which is the residue after the two-step pectin extraction, with hot water and a CDTA solution, and in the residue after the single-step nitric acid extraction (pH 1.6), i.e., the AcUF, were very similar, around 23 mol%. However, because of the harsher conditions during acid extraction, the extracted pectin was richer in RG-I domains and was characterized by a lower extent of branching due to acid hydrolysis of the neutral side chains. When the complete stepwise extraction was performed, i.e., including low-alkaline conditions in the last step, the residual fraction contained only a very low amount of UA + Rha, namely, 6 mol%. 

The disruptive effect of HPH on the AcUF improved pectin solubilization and extractability, as around two thirds of the pectin in the AcUF were solubilized or acid-extractable after HPH. However, the residual UA + Rha content present in the AcUF—HPH—AcUF was clearly higher (10 mol%) than for the lAUF, indicating that by breaking low-alkali-labile bounds, more efficient solubilization of this strongly interacting pectin fraction is possible than by mechanical disruption by HPH and a general acid extraction. After extraction with a low-alkaline medium as well as after the water and acid extraction facilitated by the physically disruptive force of HPH, the extracted pectin was shown to be richer in RG-I domains and to have a lower extent of branching. Furthermore, a higher MM of pectin seemed to be related with stronger interaction. In comparison to the average MM of the pectin present in the other EFs, the average MM of the pectin molecules solubilized by HPH was high, which indicates that HPH facilitated the release of long pectin molecules from the pectin-depleted tomato CWM in a more intact way. 

Apart from UA, the most prominent monosaccharides in the AIR of processing tomato were Glc, Xyl and Man, which can be mainly attributed to cellulose and hemicellulose. The contribution of these polysaccharides in the AIR was estimated to be approximately 56 mol%. By performing the extensive pectin extraction procedures presented in the current study, this value increased to 87–89 mol%. Only a very small amount of hemicellulose seemed to be co-extracted during the different steps of the pectin extraction procedures, even after application of HPH to the AcUF. 

As the current results allow comparing the efficiencies of different pectin extraction methods and the resulting structural properties of the extracted pectin, this study could serve in making a deliberate choice of extraction conditions when a pectin fraction of (processing) tomato with specific structural properties is desired. In the context of research on functional properties of pectin, e.g., as a gelling, emulsifying or emulsion-stabilizing agent [75], related to structure, it could be relevant to choose the appropriate extraction condition(s) to obtain pectin with the desired structural properties. For example, when the extraction of RG-I-rich pectin from tomato is aimed, harsher extraction conditions are required to disturb the stronger interactions of this pectin fraction in cell walls of processing tomato. The results of this study showing a more complete pectin extraction after HPH could be a starting point to further investigate the potential of HPH in reaching a more efficient pectin extraction from processing tomato or other fruits and vegetables than by conventional acid extraction. Furthermore, the compositional analysis of the UFs showing especially the relation between the pectin extraction method and the extent of pectin depletion could be relevant in the context of further research on possible applications, e.g., as a texturizing agent, of the (partially) pectin-depleted processing tomato CWM obtained as a side stream of pectin extraction.

## Figures and Tables

**Table 1:** Monosaccharide content (mg/g), degree of methyl esterification (DM) (%) and average molecular mass (MM) (kDa) of the extractable fractions (EFs) of processing tomato ± standard deviation (n = 2.2); < d.l. = below detection limit; n.d. = not determined.

EF	Monosaccharide Content (mg/g)							DM (%)	MM (kDa)
	Rha	Ara	Gal	Glc	Xyl	Man	UA		
WEF	9.8 ± 0.9	18.6 ± 1.6	23.7 ± 1.0	15.8 ± 0.9	11.4 ± 0.8	8.4 ± 0.2	573.9 ± 8.1	73.8 ± 2.7	114.8 ± 3.1
CEF	3.5 ± 0.4	5.1 ± 0.4	7.2 ± 0.6	4.7 ± 0.8	0.8 ± 0.4	1.8 ± 0.3	203.0 ± 14.2	n.d.	233.0 ± 4.8
lAEF	15.2 ± 3.0	9.8 ± 1.2	14.5 ± 1.2	0.9 ± 0.2	1.9 ± 0.5	<d.l.	400.2 ± 21.9	n.d.	451.0 ± 42.5
AcEF	16.4 ± 1.2	15.5 ± 0.2	19.6 ± 0.5	8.9 ± 0.2	2.1 ± 0.2	1.2 ± 0.3	558.5 ± 31.6	66.0 ± 3.6	270.3 ± 10.1
AcUF—HPH—WEF	29.3 ± 2.0	8.2 ± 0.5	19.3 ± 1.2	2.8 ± 0.5	7.3 ± 0.5	<d.l.	680.5 ± 49.6	60.5 ± 0.9	1507.5 ± 85.4
AcUF—HPH—AcEF	24.0 ± 0.9	5.4 ± 1.1	16.5 ± 1.4	4.6 ± 0.7	4.5 ± 0.2	0.9 ± 0.7	560.8 ± 46.5	47.0 ± 2.1	509.5 ± 52.8

**Table 2:** Compositional and structural properties ± standard deviation of the extractable fractions (EFs) based on the monosaccharide composition (n = 2.2). HG = homogalacturonan; RG-I = rhamnogalacturonan I.

EF	Contribution of HG (mol%)	Contribution of RG-I (mol%)	Contribution of RG-I to the Pectin Backbone (-)	Branching of RG-I (-)	Purity of the Pectin Extract (-)
	UA–Rha	2Rha + Ara + Gal	Rha/UA	(Ara + Gal)/Rha	(Rha + Ara + Gal + UA)/(Glc + Man)
WEF	83.2 ± 1.6	10.8 ± 0.5	0.020 ± 0.002	4.3 ± 0.5	24 ± 1
CEF	86.6 ± 8.3	9.9 ± 0.6	0.021 ± 0.003	3.4 ± 0.4	32 ± 4
lAEF	84.9 ± 6.6	14.3 ± 1.7	0.045 ± 0.009	1.6 ± 0.3	387 ± 90
AcEF	85.2 ± 6.7	12.6 ± 0.6	0.035 ± 0.003	2.1 ± 0.2	57 ± 4
AcUF—HPH—WEF	85.1 ± 8.8	13.3 ± 0.9	0.051 ± 0.005	0.9 ± 0.1	244 ± 45
AcUF—HPH—AcEF	85.1 ± 10.0	13.0 ± 0.9	0.051 ± 0.005	0.9 ± 0.1	105 ± 20

**Table 3:** Monosaccharide content (mg/g) of the alcohol-insoluble residue (AIR) and unextractable fractions (UFs) of processing tomato ± standard deviation (n = 2.2).

AIR or UF	Monosaccharide Content (mg/g)						
	Rha	Ara	Gal	Glc	Xyl	Man	UA
AIR	6.3 ± 0.1	12.4 ± 1.1	14.1 ± 1.0	293.1 ± 13.8	34.9 ± 2.2	26.4 ± 1.8	262.1 ± 12.9
CUF	6.6 ± 0.5	12.9 ± 1.1	16.9 ± 1.3	406.9 ± 31.3	52.2 ± 5.1	39.8 ± 2.2	163.4 ± 11.6
lAUF	2.3 ± 0.4	12.4 ± 1.3	16.3 ± 1.9	471.6 ± 17.5	58.7 ± 1.7	44.7 ± 1.3	40.2 ± 2.8
AcUF	3.9 ± 0.6	6.0 ± 1.0	14.0 ± 1.1	441.5 ± 24.5	55.6 ± 2.6	43.2 ± 2.3	182.9 ± 6.3
AcUF—HPH—AcUF	1.6 ± 0.3	2.8 ± 0.7	14.3 ± 1.8	509.6 ± 31.1	63.9 ± 4.3	52.5 ± 2.5	79.4 ± 2.2

**Table 4:** Compositional properties ± standard deviation of the alcohol-insoluble residue (AIR) and unextractable fractions (UFs) based on the monosaccharide composition (n = 2.2).

AIR or UF	Contribution of the Pectin Backbone (mol%)	Contribution of Mannans to Hemicellulose (-)	Ratio of Typical Hemicellulose Monosaccharides to Glucose (-)	Contribution of Hemicellulose and Cellulose (mol%)
	UA + Rha	Man/Xyl	(Xyl + Man)/Glc	Glc + Xyl + Man
AIR	39.0 ± 2.2	0.63 ± 0.06	0.23 ± 0.02	56.4 ± 2.6
CUF	22.7 ± 1.9	0.64 ± 0.07	0.25 ± 0.03	72.7 ± 5.4
lAUF	6.1 ± 0.4	0.63 ± 0.03	0.24 ± 0.01	89.2 ± 3.3
AcUF	23.3 ± 1.1	0.65 ± 0.05	0.25 ± 0.02	73.9 ± 3.9
AcUF—HPH—AcUF	10.3 ± 0.5	0.69 ± 0.06	0.25 ± 0.02	87.3 ± 5.3

## Data Availability

The data are contained within the article.

## Abbreviations

The following abbreviations were used in the manuscript.

AcEF	acid-extractable fraction
AcUF	acid-unextractable fraction
AcUF—HPH—AcEF	acid-extractable fraction of the acid-unextractable fraction subjected to HPH
AcUF—HPH—AcUF	acid-unextractable fraction of the acid-unextractable fraction subjected to HPH
AcUF—HPH—WEF	water-extractable fraction of the acid-unextractable fraction subjected to HPH
AIR	alcohol-insoluble residue
Ara	arabinose
CDTA	cyclohexane-trans-1,2-diaminetetraacetic acid
CEF	chelator-extractable fraction
CUF	chelator-unextractable fraction
CWM	cell wall material
DM	methyl esterification
EDTA	ethylenediaminetetraacetic acid
EF	extractable fraction
FT-IR	Fourier-transform infrared
Fuc	fucose
Gal	galactose
GalA	galacturonic acid
Glc	glucose
GlcA	glucuronic acid
HG	homogalacturonan
HPAEC–PAD	high-performance anion exchange chromatography with pulsed amperometric detection
HPH	high-pressure homogenization
lAEF	low-alkaline-extractable fraction
lAUF	low-alkaline-unextractable fraction
Man	mannose
MM	molecular mass
RG-I	rhamnogalacturonan I
RG-II	rhamnogalacturonan II
Rha	rhamnose
UA	uronic acid
UF	unextractable fraction
WEF	water-extractable fraction
Xyl	xylose
